# Puzzling onsets of pneumonia sequentially after each session of bronchial thermoplasty: a case report

**DOI:** 10.1186/s12890-020-01243-2

**Published:** 2020-08-11

**Authors:** Ying Nong, Jiang-tao Lin

**Affiliations:** Department of Pulmonary and Critical Care Medicine, China-Japan Friendship Hospital, Beijing, China

**Keywords:** Asthma, Bronchoscopy, Pneumonia, Case report

## Abstract

**Background:**

Bronchial thermoplasty (BT) is a novel bronchoscopic intervention for severe persistent asthma. An increase in transient respiratory adverse events associated with BT were noted over the treatment periods, however, these events appear sporadic and should not always recur in a single individual and BT-related pneumonia has rarely been reported.

**Case presentation:**

We present a case of uncontrolled severe asthma who developed puzzling pneumonia sequentially after each session of BT procedures. After each operation of three sequential BT procedures, she developed cough and purulent expectoration when her chest radiology showed new infiltrates right in the treatment regions. After empirical use of antibacterial agents plus physiotherapy and postural sputum drainage, her symptoms vanished and chest imaging resumed normal.

**Conclusion:**

The originality of our case report is related to the recurrence of pneumonia after three sequential BT procedures. To date, similar report has not been available in the literature. We hope to prompt alerts for post-BT respiratory infections, although most of them, along with other adverse events, are mild and tractable.

## Background

For severe cases of asthma, bronchial thermoplasty (BT) is a novel bronchoscopic intervention, proposed to reduce airway smooth-muscle mass, airway hyperresponsiveness and bronchoconstriction [1]. A typical BT is performed over three bronchoscopy sessions, each by 3–4 weeks apart to address first the right lower, then the left lower, and finally both upper (including the lingular) lobes of the lungs. Clinical studies have demonstrated the benefits of BT in favor of asthma control, severe exacerbation, quality of life, medication use, medical costs, and therapeutic efficacy [1–5].

Although the long-term efficacy and safety of BT have been validated [6], an increase in transient respiratory adverse events were also noted over the treatment periods, including cough, wheezing, lower respiratory infection, recurrent atelectasis, and hemoptysis [7–11]. However, these events appear sporadic and should not always recur in a single individual and BT-related pneumonia has rarely been reported. Here, we describe a female with uncontrolled severe asthma who developed puzzling pneumonia sequentially after each session of BT procedures. To date, similar report has not been available in the literature.

## Case presentation

**Fig. 1:**
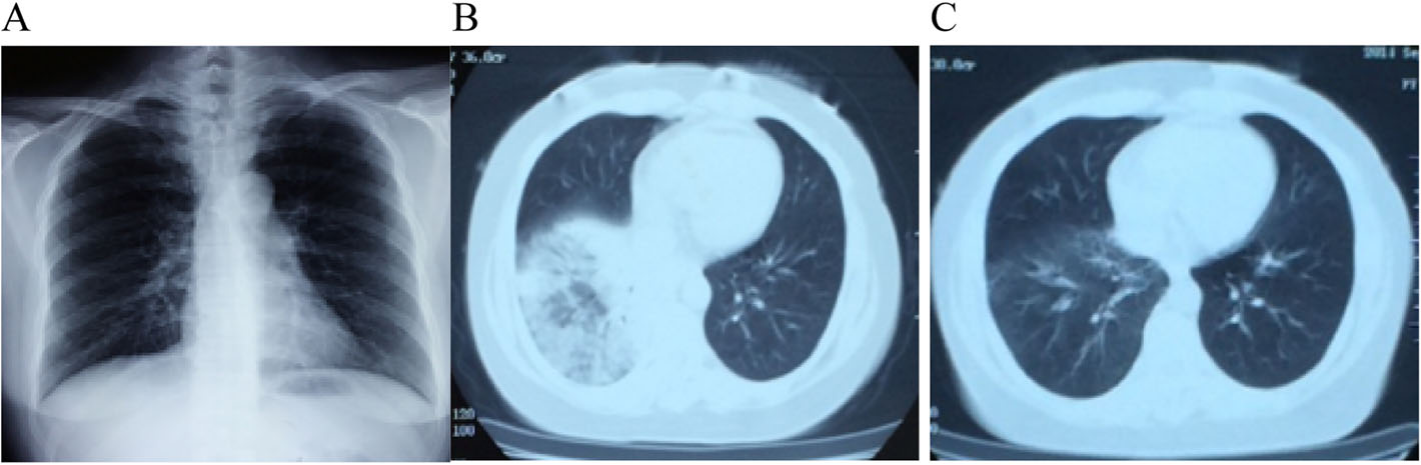
Chest images before or after the first BT session. **a** Chest X-ray was normal before the right lower lobe BT treatment. **b** Chest CT revealed infiltrative shadows in the right lower zone with air bronchogram sign after the right lower lobe BT procedure. **c** Chest CT showed the infiltrates in the right lower lobe had significantly resolved after one-week of antibacterial treatment

A 36-year-old female presented with a 3-year history of wheeze and had been diagnosed with severe asthma, allergic rhinitis and chronic sinusitis. Despite of high-dose inhaled corticosteroids and long-acting beta_2_-agonist, along with oral prednisone, her asthma had been uncontrolled and had acute exacerbations nearly once monthly. She was then referred to our institution for BT. Prior to the first session, her baseline white blood cell count was 6.63 × 10^9^/L, with eosinophils of 0.01 × 10^9^/L and neutrophils of 80.2%. Erythrocyte sedimentation rate (ESR), C-reactive protein (CRP) and chest X-ray (Fig. 1a) were normal. Pulmonary function test showed severe airway obstruction with forced expiratory volume in 1 s (FEV_1_) of 1.25 L (46.8% predicted) and forced expiratory volume in 1 s/forced vital capacity (FEV_1_/FVC) of 57.08%. Induced sputum was eosinophilic (62%; normally < 3.0%) and fractional exhaled nitric oxide (FeNO) test read 58 ppb. Following the protocol for perioperative management [12], she was given 30 mg prednisone daily for 5 days. On the day of procedure, her FEV_1_ had raised to 2.60 L (97.4% predicted), and FEV_1_/FVC to 80.25%.

The patient underwent the first session of standard BT according to the wide-accepted Chinese guidelines with 87 effective activations delivered to the right lower lobe [12]. During the procedure, the airways were noted to be clear with normal appearance on bronchoscopy. On the next day, the patient developed purulent sputum, mild wheezing after exercise, but was afebrile. She was discharged home, prescribed with oral prednisone 30 mg daily. On day 14 off the first BT session, the patient developed fever (38.7 °C) and coughed up massive yellow sputum. She was found to have high CRP level (100 mg/dL, normally < 0.8 mg/dL), elevated white blood cells (11.3 × 10^9^/L) and neutrophils (8.71 × 10^9^/L) counts, and normal eosinophils. Chest computed tomography (CT) revealed pulmonary infiltrates in her right lower lobe with air bronchogram signs (Fig. 1b). Based on the clinical diagnosis of pneumonia, a one-week treatment with Moxifloxacin 400 mg IV daily was started, which resulted in recovery of her normal temperature, significantly less sputum, and a peak expiratory flow rate (PEF) of 340 L/min (personal best, 390 L/min). The infiltrates in the right lower lobe had resolved on follow-up CT (Fig. 1c). Prednisone was reduced to 20 mg per day.

**Fig. 2:**
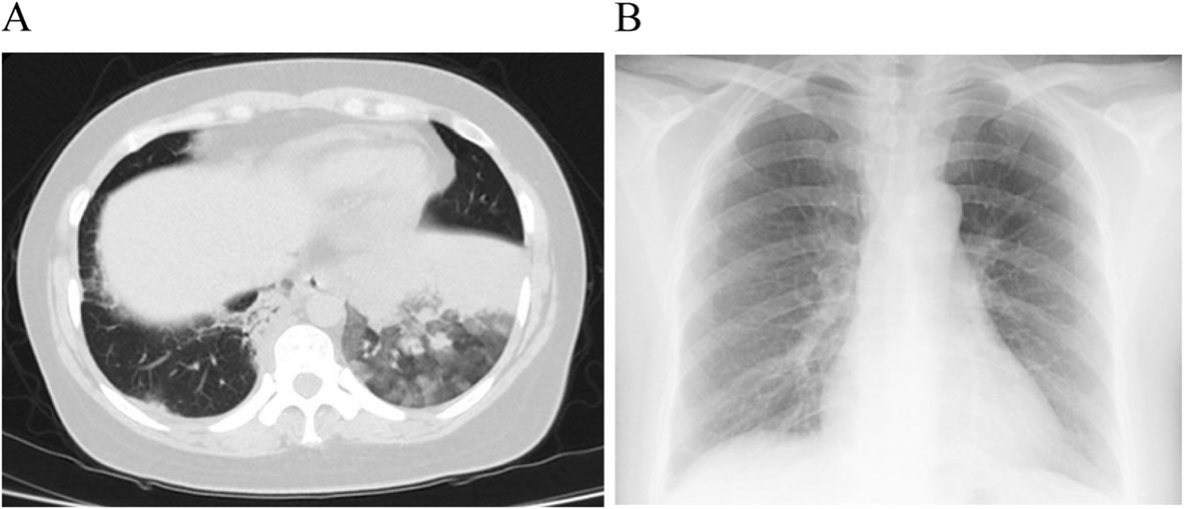
Chest images after the second BT session. **a** chest CT showed new infiltrates in her left lower lobe after the left lower lobe BT treatment. **b** Chest X-ray showed the infiltrates in the left lower lobe had disappeared after five-day of antibacterial treatment

Prior to the second BT session on day 26 off the first one, the patient presented normal white blood cells (9.38 × 10^9^/L) with 73.5% neutrophils, normal CRP (0.421 mg/dL) and FeNO (25 ppb). Her lung function was better with FEV_1_ of 2.67 L (101.9% predicted), and FEV_1_/FVC of 81.93%. The second BT session delivered 102 effective activations to her left lower lobe. Under bronchoscopy, a considerable amount of white sputum in the right main bronchus was removed by suction, while the bronchial mucosa in the lower left lobe appeared hyperemic with scarce mucus lining. Given her pneumonia in the first session, prophylactic Moxifloxacin 400 mg IV once daily was prescribed immediately after BT. Still, the patient complained of cough, yellow sputum without fever and wheezing 2 days later. One week post-BT, although with normal hemogram (WBC 8.08 × 10^9^/L, neutrophils 3.31 × 10^9^/L) and ESR (8 mm/h), chest CT showed new infiltrates in her left lower lobe (Fig. 2a). No specific pathogens were identified from bronchial mucus and sputum cultures. The anti-infective treatment was escalated to Cefoperazone/sulbactam 2 g IV q12h plus Moxifloxacin 400 mg IV once daily, along with physiotherapy and postural drainage to facilitate sputum expectoration. These led to relief on her coughs and sputum, and resolution of the lobar infiltrates on chest X-ray 5 days later (Fig. 2b). She was discharged with the dose of prednisone reduced to 15 mg daily. After one more week on oral moxifloxacin, she had no more cough, sputum, or wheezes and her PEF elevated to 400 L/min.

**Fig. 3:**
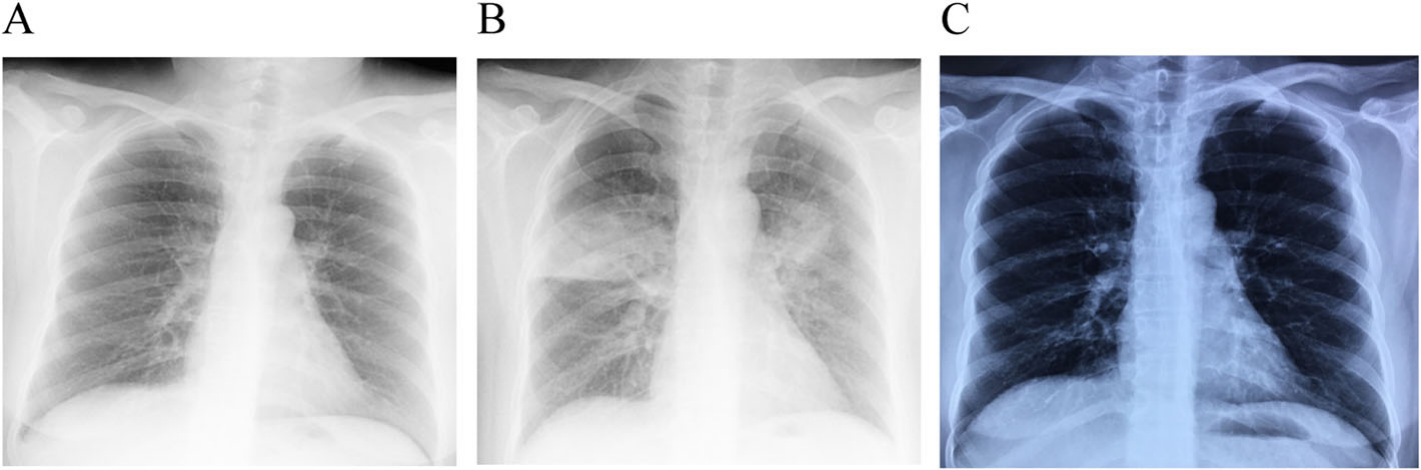
Chest X-ray before or after the final BT session. **a** There was no remarkable abnormality on chest X-ray before the third session of BT. **b** Chest X-ray revealed infiltrates newly developed in both upper lobes after both upper lobes BT procedure. **c** Chest X-ray showed the infiltrates in both upper lobes had diminished after 10-day of antibacterial treatment

The third and final BT session was scheduled on day 24 off the second one. On admission, she had a white blood cell count of 5.56 × 10^9^/L with 58.3% neutrophils, a CRP of 0.342 mg/dL, an ESR of 7 mm/h, and an FeNO of 18 ppb. Lung function test showed FEV_1_ of 2.57 L (96.6% predicted) and FEV_1_/FVC of 81.07%. There was no remarkable abnormality on chest X-ray (Fig. 3a). In this session, 105 activations were delivered to her both upper lobes. Bronchoscopic inspection did not reveal any unusual signs in the bronchial lining. Surprisingly, coughs and purulent expectoration without fever, developed on the next day, regardless of her hemogram, ESR and CRP remaining within normal range on a second check. Chest X-ray revealed infiltrates newly developed in her both upper lobes (Fig. 3b). Sputum culture was again negative. As with the previous episode, the same dosing of IV Cefoperazone/sulbactam plus Moxifloxacin were administrated empirically for 10 days, with prednisone reduced to 10 mg daily. Ten days later, the patient experienced dramatical relief in symptoms with PEF up to 400 L/min. Chest X-ray showed the infiltrates in both upper lobes had diminished (Fig. 3c). Thereafter and during the two-year follow up, the patient did not develop pneumonia and showed good control of asthma. She reported no hospitalization due to acute exacerbation and completely withdrawed oral corticosteroids. Her lung function had been stable over the 2 years after BT.

## Discussion and conclusions

Although the 5-year safety profiles of BT have been documented [6], certain adverse events associated with BT during or shortly after the procedure have been noted [7–11]. Given the skilled expertise of manipulators and rigorous practice of sterile principle in special institution, these events appear sporadic and should not always recur in a single individual. Furthermore, BT-related pneumonia has rarely been reported.

Matsubayashi S. et al. described a case of *Aspergillus fumigatus* and *Nocardia spp*. infections that developed after the first and second BT procedures [10]. They attributed the resultant infection to systemic use of steroids which might immunocompromise the patient, and to the facilitated tissue fragility caused by thermal energy from BT. Balu A. et al. reported a 43-year-old female who developed a lung abscess as an immediate complication 3 days after the second BT session [8], most likely associated with the transient localized airway obstruction due to BT. In the AIR2 trial, one patient in the BT arm needed hospital admission due to lower respiratory tract infection [3], but detailed information about the infection was not shown. To the best of our knowledge, the case presented herein is the first-ever to report on pneumonia localized in the treatment region after each of the three sequential BT procedures. It was puzzling that although the patient presented coughs and yellow sputum after each BT session, typical manifestations of an infection (such as fever, elevated white blood cells, ESR and CRP) were evidenced after the first BT session only. She persisted to be afebrile and with normal hemograms after the other two sessions, while chest radiology showed new infiltrates right in the regions corresponding to treatment targets. One possible explanation is that the patient could have had a prior airways colonization and exacerbated by the procedure, but unfortunately, we did not get any positive bronchoalveolar lavage fluid or sputum culture results. Despite negative microbiological tests, her symptoms vanished and chest imaging resumed normal after empirical use of antibacterial agents plus physiotherapy and postural sputum drainage.

It remains unclear why the patient developed pneumonia exactly after each BT treatment. The use of high-dose inhaled or oral corticosteroids might have immunocompromised her and made her more susceptible to infections. In addition, the mucosal tissue fragility and unpredictable airway responses [13] (eg. bronchoconstriction, edema, mucus hypersecretion, mucosal bleeding) together with the formation of fibrin-rich plugs in the airways [11] secondary to BT might affect the airway secretion drainage and lead to segmental atelectasis and infection. However, these scenarios can be present in a number of patients undergoing BT, and do not explain exclusively for the repeated onsets of pneumonia in our case; moreover, as we noticed in each session, there were not much mucus secretion in her targeted bronchi.

There was evidence that the number of activations per procedure may be a factor in the immediate fall in FEV_1_ 24 h after BT [14]. On the other hand, the number of activations has a role in determining the clinical response to treatment [15]. By using a thinner bronchoscope, greater number of radiofrequency activations deployed were not associated with an increase in post-procedural adverse event [16]. Yamamoto S. et al. also revealed that increase in the number of radiofrequency activations during BT is related to a decrease in FEV_1_ and %FEV_1_ from baseline. The number of radiofrequency activations, however, is not associated with SAEs including pneumonia after BT [17]. The patient presented herein received a total of 294 effective activations which were more than the average activations in practice [13]. We cannot determine whether the more BT activations caused pneumonia in this patient. Future studies are warrant to address this relationship.

In this case report, we presented an unusual case of pneumonia that developed in the corresponding treatment area after each of three sequential BT procedures. Although most adverse respiratory events post-BT are transient and tractable, alerts should be prompted for onsets of pneumonia in certain patients who are on long-term use of corticosteroids or those with severe airway remodeling as reflected by poor lung function and uncontrolled symptoms. Premedications aiming to reduce mucus secretion and facilitate sputum expectoration, bronchial mucus sampling for microbiological test, a reasonable number of activations, and as-needed antibiotics for patients with severely impaired lung function or mucus hypersecretion should be beneficial to secure a satisfactory outcome of BT.

## Data Availability

The datasets used and/or analyzed during the current study are available from the corresponding author on reasonable request.

## Abbreviations

BT: Bronchial thermoplasy
CRP: C-reactive protein
ESR: Erythrocyte sedimentation rate
FEV_1_: Forced expiratory volume in one second
FVC: Forced vital capacity
FeNO: Fractional exhaled nitric oxide
IgE: Immunoglobulin E
CT: Computed tomography
PEF: Peak expiratory flow
